# Pulmonary Embolism in Women: A Systematic Review of the Current Literature

**DOI:** 10.3390/jcdd9080234

**Published:** 2022-07-25

**Authors:** Rosy Thachil, Sanjana Nagraj, Amrin Kharawala, Seth I. Sokol

**Affiliations:** NYC Health + Hospitals/Jacobi Medical Center and the Albert Einstein College of Medicine, 1400 Pelham Parkway S, The Bronx, NY 14061, USA; nagrajs@nychhc.org (S.N.); kharawaa@nychhc.org (A.K.); seth.sokol@nychhc.org (S.I.S.)

**Keywords:** sex-differences, pulmonary embolism, gender-differences, women’s health, women’s cardiac health equity

## Abstract

Cardiovascular disease is the leading cause of death in women. Pulmonary embolism (PE) is the third most-common cause of cardiovascular death, after myocardial infarction (MI) and stroke. We aimed to evaluate the attributes and outcomes of PE specifically in women and explore sex-based differences. We conducted a systematic review of the literature using electronic databases PubMed and Embase up to 1 April 2022 to identify studies investigating PE in women. Of the studies found, 93 studies met the eligibility criteria and were included. The risk of PE in older women (especially >40 years of age) superseded that of age-matched men, although the overall age- and sex-adjusted incidence of PE was found to be lower in women. Risk factors for PE in women included age, rheumatologic disorders, hormone replacement therapy or oral contraceptive pills, pregnancy and postpartum period, recent surgery, immobilization, trauma, increased body mass index, obesity, and heart failure. Regarding pregnancy, a relatively higher incidence of PE has been observed in the immediate postpartum period compared to the antenatal period. Women with PE tended to be older, presented more often with dyspnea, and were found to have higher NT-proBNP levels compared to men. No sex-based differences in in-hospital mortality and 30-day all-cause mortality were found. However, PE-related mortality was higher in women, particularly in hemodynamically stable patients. These differences form the basis of future research and outlets for reducing the incidence, morbidity, and mortality of PE in women.

## 1. Introduction

Pulmonary embolism (PE) is the third most-common cause of cardiovascular death after myocardial infarction and stroke [1]. Based on epidemiological research conducted using CDC data in the US, between 1999 and 2018, 159,572 deaths were attributed to PE [2]. Additionally, the incidence of venous thrombosis and PE as per the European guidelines is found to be approximately 0.5–1 per 1000 [3]. PE is considered a great masquerader, as it may present with a wide spectrum of symptoms, many of which may be shared with other clinical diagnoses; this can make definitive diagnosis challenging. As a result, special attention should be given to factors such as age, risk factors, and even sex-based differences in the presentation and management of PE. Most of the existing cardiovascular literature is centered around male patient populations. Recent studies, however, have started to explore sex-based differences in the presentation and management of cardiopulmonary conditions. It has been established that women tend to present with atypical symptoms of myocardial infarction, which leads to a delay in diagnosis, lower rates of PCI, and higher mortality as compared to men [4,5]. Sex-based differences have also been observed in risk factors, presentation, management, and outcomes of other cardiovascular conditions including stroke, carotid stenosis, heart failure, and abdominal aortic aneurysm [6].

In addition to reviewing the literature for sex-based differences in the incidence, risk factors, clinical features, diagnosis, prognosis, mortality, and complications of PE, we also aimed to address PE in pregnancy and COVID-19. The changes in sex hormones, difference in prevalence of vasculopathies, physiological changes in pregnancy and post menopause, and use of oral contraceptive pills (OCPs) or hormone replacement therapy (HRT) are a few pathophysiological mechanisms which have been traditionally used to explain sex-based differences surrounding coronary artery disease (CAD) [5]. Similar to HRT, pregnancy can lead to a pro-thrombotic state, resulting in venous thrombosis and subsequent PE [7]. However, studies done on the venous side of the vasculature are few and inconclusive, especially in correlating sex-based differences in PE. We also sought to address COVID-19 and its relationship to women and PE, as increased incidence of PE has been found in patients with SARS-CoV-2 virus, with an estimated mean incidence of 7.4% according to a Cochrane review [8]. Due to its current relevance to healthcare and cardiovascular care, it is crucial to understand COVID-19’s behavior and effects in both sexes. Through this systematic review, we aimed to explore the epidemiology, presentation, management, and outcomes for PE in women as it is understood in the literature to date.

## 2. Materials and Methods

We conducted a systematic review of the literature using electronic databases PubMed and Embase from inception to 1 April 2022 to identify published studies evaluating pulmonary embolism in women and sex-based differences pertinent to PE. The combination of keywords used was ‘pulmonary embolism’ AND (‘sex differences’ or ‘sex-based’ or ‘gender differences’ or ‘gender-based’ or ‘sex disparities’ or ‘sex distribution’ or ‘sex characteristics’ or ‘sex dimorphism’) or (‘pulmonary embolism [ti] (title)’) AND (‘female [ti]’ or ‘females [ti]’ or ‘women [ti]’). A separate search of the electronic database PubMed was conducted to evaluate the role of COVID-19 in PE in women using an exclusive search strategy independent of the one described earlier. The specific keywords used were ‘pulmonary embolism’ AND (‘sex differences’ or ‘sex-based’ or ‘gender differences’ or ‘gender-based’ or ‘sex disparities’ or ‘sex distribution’ or ‘sex characteristics’ or ‘sex dimorphism’) AND (‘COVID-19’ or ‘SARS-CoV-2’ or ‘coronavirus’). The results of sex-based differences in patients with PE and COVID-19 were evaluated separately and presented in Section 3.9.2. Regarding study protocol registration, we did not register our study prospectively given the significant processing delays.

### 2.1. Eligibility Criteria

A study was included in this systematic review if it fulfilled the following criterion: peer-reviewed prospective or retrospective analysis that evaluated pulmonary embolism in women and/or assessed sex-based differences in PE attributes. All identified articles were assessed against the following exclusion criteria: studies with no human subjects; lack of quantitative analysis of variables of interest; and studies not published as full texts in English language. The same eligibility criteria were used while evaluating the role of COVID-19 in women presenting with PE, with the addition of serological confirmation of COVID-19, which was required for study inclusion.

### 2.2. Data Extraction

**Figure 1:**
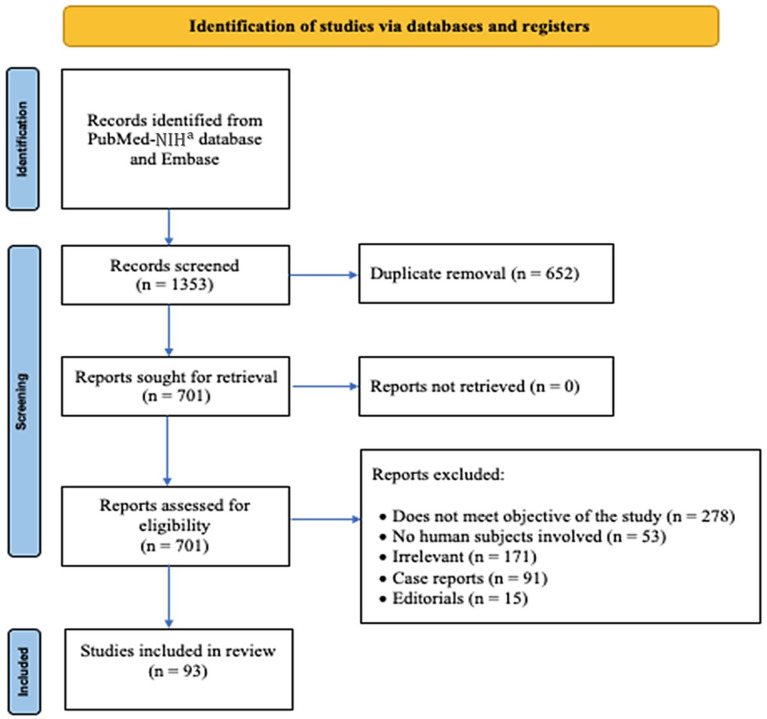
PRISMA flow diagram. PRISMA: Preferred Reporting Items for Systematic Reviews and Meta-Analyses. ^a^ National Institutes of Health.

Studies were screened for eligibility and selected using the Preferred Reporting Items for Systematic Reviews and Meta-Analyses (PRISMA) guidelines (PRISMA checklist in Table S1 & PRISMA flow diagram in Figure 1) [9]. Reference lists of all eligible studies were reviewed to identify additional studies. After manually excluding duplicate and irrelevant studies, data were extracted from each eligible study by authors SN and AK, who were blinded to each other. Study designs and data from each of the potentially eligible studies were scrutinized again for comparability by these authors. Disagreements were resolved by discussion, and a final decision was reached by consensus with the addition of a third reviewer RT.

## 3. Results

### 3.1. Search Results

**Figure 2:**
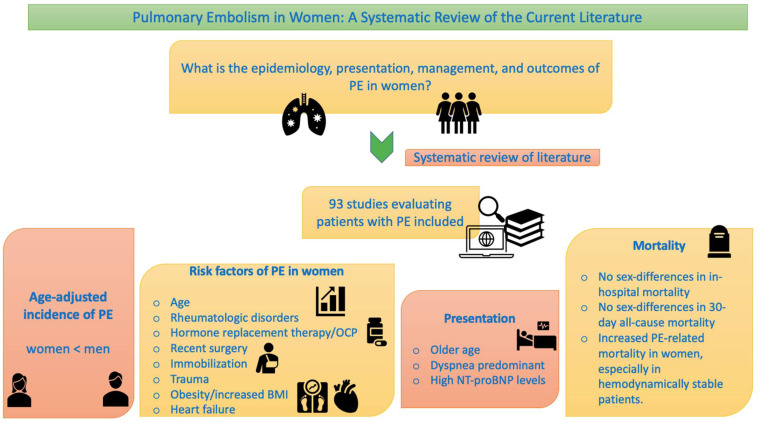
Characteristics of pulmonary embolism in women.

The literature search yielded 1353 studies, of which 701 studies were retrieved for full-text evaluation after duplicates were removed. 278 studies were excluded as they did not meet the objective of study as outlined in the Section 2.1 “eligibility criteria”, i.e., these studies did not evaluate pulmonary embolism in women and/or assess sex-based differences in one or more attributes of PE. Of the studies, 53 studies did not involve human subjects, 171 studies were irrelevant and unrelated to PE, 91 studies were case reports, and 15 were editorials. At the end of the review process, 93 studies fulfilled the predetermined inclusion criteria and were included in our systematic review, as shown in the PRISMA flow diagram (Figure 1). A visual summary of characteristics of PE in women is presented in Figure 2.

### 3.2. Epidemiology

#### 3.2.1. Incidence

In the general adult population, the age-adjusted incidence of PE appears to be around 30–100 cases per 100,000 person-years [10,11,12,13] while the age- and sex-adjusted incidence of PE appear to be around 69 per 100,000 [11]. The incidence of pulmonary embolism increases with age in both sexes [10,11,14,15], with a relative risk (RR) of venous thromboembolism (VTE) that nearly triples between 60–69 years of age and at >80 years of age (RR: 5.983; 95%CI: 5.708–6.273; *p* = 0.0001) vs. RR: 14.890; 95%CI: 14.103–15.721; *p* = 0.0001). Although a review by Jarman et al. reports a similar overall age-adjusted incidence of PE between the sexes, most observational studies and reviews in the literature have found men to have a higher overall age-adjusted incidence of PE compared to females [10,11,16,17,18], in a male:female sex ratio of 1.2:1 [16].

Despite a higher age-adjusted incidence of PE in men, the patterns of developing PE vary with age among women and men [14,18,19]. Studies have suggested that women diagnosed with PE are more likely to be older than men [15,20,21,22]. In the 4-year study conducted by Choi et al. evaluating sex differences in the incidence of PE in hospitalized patients, in patients > 50 years of age, PE was more frequent in women (incidence: 0.15%; 95%CI: 0.11–0.19%) compared to men (incidence: 0.08%; 05%CI: 0.05–0.11%; *p* < 0.01) [23]. Similarly, in another study from Japan evaluating the incidence of PE, in patients under 40 years of age, more men developed PE, but in all age groups >40 years of age, a significantly higher number of women developed PE compared with men [24].

Men have also been found to have a higher incidence of recurrent pulmonary embolism [25]. In the multicenter registry by Verso et al. assessing long-term outcomes of VTE, male sex was found to be an independent risk factor for recurrent VTE [25,26]. Similarly, Tagalakis et al. found men to have a significantly higher rate of recurrent PE compared to women (adjusted HR (hazard ratio): 1.13; 95%CI: 1.07–1.19) over a mean follow-up period of 3.9 years [26]. The investigators found a small but significant difference in the cumulative 5-year probability of recurrent VTE between the sexes (12.4% in men vs. 10.9% in women; *p* = 0.0001) [26]. Of note, despite a higher incidence of recurrent PE and overall age-adjusted incidence of PE, men with acute PE were, irrespective of whether first time or recurrent, were more likely to be taking an antiplatelet medication at the time of presentation [20].

#### 3.2.2. Risk Factors for PE

Several comorbidities and patient characteristics have been linked to the development of PE in women. As discussed above, increased age is a well-established risk factor for PE in women. Multiple studies have shown that women with PE were more likely to have a diagnosis of rheumatologic disorders (6.1% of women vs. 3.3% of men; *p* < 0.01) compared to men [20,27,28,29]. Specifically, systemic lupus erythematosus and rheumatoid arthritis (RA) have been found to be significant risk factors for PE after adjusting for age, sex, and other comorbidities. In a large nationwide study of 23.7 million individuals from China, 29,238 had RA, of whom the overwhelming majority was female (77%), and the risk of developing PE was more than double in those with RA compared to those without [27]. However, among patients with RA, no sex differences in the incidence of PE were found [27].

Oral contraceptive pills (OCP) and hormone replacement therapy, which are well-established risk factors for VTE, are more likely to be present in women who present with PE compared to men (14.8% women vs. 0.8% men; *p* < 0.001) [20,22,30]. Additionally, those on OCPs have been found to have a higher likelihood of right ventricular strain with PE compared to those who were not taking OCP (*p* = 0.003) [31]. Heart failure has also been found to be an important risk factor for PE in women [32,33,34], although men with heart failure and PE were more likely to suffer in-hospital mortality during their PE admission compared to women with heart failure and PE [33]. Among other evaluated risk factors, history of recent surgery [16,35], immobilization [16,36], and trauma have also been significantly associated with the development of PE and are more prevalent in women compared to men (38.4% women vs. 29.5% men; *p* = 0.026) [22]. Notably, in both comparative studies evaluating VTE and total joint arthroplasty, female sex was found to be a significant independent risk factor of PE for both total knee and hip arthroplasties [35,37].

Other relevant characteristics include a higher prevalence of dementia among women with PE, although there are limited data [20]. This is consistent with other studies reporting a relatively increased incidence of PE among older women compared to men of similar age. Increased subcutaneous fat, BMI (body mass index), and obesity have also been found to increase the risk of PE in women [16,38,39]. Other commonly associated risk factors include a history of smoking, prior VTE, cancer [15,22], coronary artery disease and myocardial infarction [17,20,22], renal failure [15], and severe liver disease, all of which were less prevalent in women with PE compared to men [20]. Additionally, male sex has been found to be a significant independent predictor of congenital thrombophilia in patients with PE [40], and this was consistent across all groups of hereditary thrombotic disorders, including protein C deficiency, protein S deficiency, antithrombin 3 deficiency, abnormal lupus anticoagulant, antiphospholipid syndrome, factor V Leiden, and hyperhomocysteinemia [41].

### 3.3. Clinical Features

There are notable differences in clinical features of PE in women. An observational prospective study found a similar frequency of dyspnea, chest pain, tachycardia, hypoxemia, and hypotension; however, the female sex was found to have higher rates of syncope and elevated NT-proBNP [15]. A registry-based study by Tanabe et al. in Japan found that women had a higher frequency of dyspnea, increased serum NT-proBNP (180.4 [50.7 to 526.1] pg/mL vs. 107.0 [25.0 to 306.8] pg/mL; *p* < 0.0001), and higher pulmonary arterial systolic pressure (51.5 ± 22.2 mm Hg vs. 47.4 ± 22.4 mm Hg; *p* = 0.012) [21]. Similarly, Serbian investigators also found a higher level of plasma NT-proBNP and increased incidence of acute heart failure symptoms in women; however, on multivariate analysis, the difference in the rate of acute heart failure among sexes disappeared due to a strong influence of age, as women presenting with PE were significantly older [42]. The presence of higher NT-proBNP values in women could possibly be explained by the fact that Tanabe et al. found a higher incidence (14.6% to 9.2%; *p* = 0.0002) of severe cases with massive PE in women as compared to men [21]. Additionally, women were also found to have a higher incidence of nosocomial infections, lower blood pressure, and higher respiratory rate [21]. While Pribish et al. also found a significantly high NT-proBNP level in women, they found that women were more likely to have a normal RV size on echo (63.2% vs. 54.8%; *p* = 0.01), despite a similar incidence of PE severity as compared to men [20]. This was a large single-center study conducted in the USA [20]. In contrast, a post-mortem-based study in North Carolina showed that female gender could predict the presence of a massive PE in patients with PEA arrest [43]. Notably, another study noted a higher incidence of massive PE in African-American females [44].

McHugh et al., who reviewed approximately 4500 patients in the International Cooperative Pulmonary Embolism Registry (ICOPER), found that chest pain and hemoptysis occurred more frequently in men, while re-iterating the increased frequency of dyspnea in women [45]. This was also seen across various retrospective, multicenter and registry-based studies [20,21,42,46,47]. On the contrary, some investigators did not find a significant difference in the frequency of chest pain among the sexes [15,20]. Some studies have also found a higher incidence of fever in men [42], while other studies showed equal incidence [45]. The high frequency of hemoptysis could possibly be attributed to the increased incidence of cancer in men; however, it is difficult to prove a true association. We would benefit from specific studies exploring the association of these symptoms from a sex-specific lens and correlating it with sex-specific risk factors.

Based on the current literature, it can be concluded that women tend to present at older ages and with higher NT-proBNP levels. However, the severity of presentation in women requires more dedicated research.

### 3.4. Diagnosis

It is important to understand sex-based differences in the accuracy of diagnostic tools for PE and the possible differences in diagnostic findings to help facilitate better management strategies. A study by Ebadi et al. compared the validated diagnostic predictive tools such as Well’s score and Geneva score and found no sex-based difference in the predictive characteristics of these tools across all levels of clinical probabilities [46]. This was consistent with the study by van Mens et al., who also found no sex-based differences in the predictive power of Wells rule with fixed D-dimer, Well’s rule with age-adjusted D-dimer, and YEARS algorithm [48]. Ebadi et al. also found that even though the overall prevalence of PE was equal among both sexes, men underwent more non-invasive diagnostic workup as compared to women [46]. Compression ultrasonography was more useful for ruling in, and D-dimer was more useful for ruling out PE in men as compared to women, which led to an increased number of women undergoing CTPA to achieve a final diagnosis as compared to men (64% vs. 57%; *p* = 0.001) [49]. Another ED-based study found higher utilization of CTPA in women (*p* < 0.001) without any difference in PE positivity rates in men [50]. A Netherlands-based study concluded that the yield of CTPA could be improved in women [51]. Aggarwal et al. showed that male sex was associated with a higher positivity rate on CTPA, especially within the age range of 18–35 years [52], based on which we can argue that there could be a sex-based difference in the efficacy of diagnosis of PE using a CTPA. This could possibly be attributed to the difference in clinical presentation, with men having more proximal DVTs (43% vs. 33%; *p* = 0.009) [46], women with a higher prevalence of de novo PE [53], and presence of less-obvious diagnostic signs in women. However, using data from the PIOPED II trial (NCT00007085), Stein et al. found that despite having no sex-based difference in the sensitivity of CTPA, the specificity was in fact higher in women as compared to men (97% vs. 93%; *p* = 0.015) [54]. In contrast, an imaging-based study found no influence of patient’s sex on imaging parameters [55], and data collected by Stein et al. from the National Hospital Discharge Survey did not find a sex-based difference in the diagnosis of PE, use of diagnostic tests for PE, or the duration of hospitalization for PE [56]. The PIOPED 2 study did find, however, that the diagnostic yield of non-invasive tests like D-dimer for ruling out PE was generally higher in men as compared to women [49]. It is also important to consider estrogen use in females, as it has been associated with a higher prevalence of PE with a relatively lower efficiency of validated diagnostic predictive tools and a higher D-dimer in this cohort [48]. While some studies did not find a significant difference among sexes in relation to concomitant DVT [57], other studies found more DVTs in men [20,46,49]. This could correlate to the higher frequency of calf pain found in men with PE. It was interesting to note that studies found that despite similar severity of PE among sexes (massive, sub-massive, or low risk) [20], women with acute PE were more likely to have a normal RV size (63.2% vs. 54.8%; *p* = 0.01), while men had RV enlargement [20]. Yet, paradoxically, they found women to have higher NT-proBNP as mentioned above [20]. There were no sex-based differences in PE location, computed tomography (CT) evidence of right heart strain, RV function on surface echocardiogram, or troponin elevation [20]. Based on a Serbian study, RVSP on echo, embolic burden score on MDCT-PA, and frequencies of typical EKG signs were similar among both sexes [42]. Jenab et al. compared the tissue doppler parameters and found that even though the values for parameters like tricuspid annular plane systolic excursion or right ventricular (RV) peak systolic strain were not initially different among sexes, the overall improvement in these parameters was faster in men as compared to women [58]. They also concluded that the midventricular peak systolic strain could be useful to monitor the recovery process [58].

### 3.5. Management

Historically, there has been a tendency to undertreat women for cardiovascular disease. For example, in myocardial infarction (MI), there was a significant disparity in treatment between sexes, which resulted in lower rates of primary PCI and aspirin usage and longer door-to-balloon times that resulted in increased in-hospital mortality and further complications in women with STEMI [59]. In recent years, with more advances in knowledge and technology, there has been a push towards health equity, with a significant improvement in equalization of treatment opportunities, resulting in similar outcomes between sexes in procedures like TAVR [60] and Mitraclip [61]. For PE, the standard management includes treatment with anticoagulation in hemodynamically stable patients, while thrombectomy and catheter thrombolysis are reserved for patients with severe or massive PE or those with massive PE burden who are unable to tolerate thrombolysis or AC [62].

In trying to understand the sex-based differences in the management of PE, Keller et al. conducted a prospective single-center study of 569 patients and found that despite similarity in PE-related diagnostic studies and PE severity, women were more often treated with systemic thrombolysis (16.4% vs. 9.2%; *p* = 0.013), while no sex-based difference was seen in embolectomy [22]. They also found that the relative risk of an adverse outcome in patients with high and intermediate-high risk PE was more significantly reduced by the use of reperfusion therapy in women (reduction from 39.09; 95%CI: 9.29–164.40; *p* < 0.001 to 23.43; 95%CI: 5.44–101.00; *p* < 0.001) as compared to men (reduction from 5.78; 95%CI: 2.57–12.98; *p* < 0.001 to 2.69; 95%CI: 1.07–6.78; *p* = 0.036) [22]. However, they found higher rates of major bleeding in women, with major bleed being a significant predictor of all-cause mortality only in women [22]. Interestingly, a study analyzing the National Inpatient Sample found that as the use of thrombolysis increased between 2006 and 2011 in the USA, it was seen that more white men living in higher-income ZIP codes underwent treatment with thrombolysis as compared to the female sex (OR (odds ratio): 0.78; 95%CI: 0.75–0.81; *p* < 0.01) [63]. This sex-based difference could possibly be attributed to the fact that women tend to experience more bleeding with thrombolytics as compared to men [15,22,64]. It could also be explained by the large MAPPET study, which showed that in men with sub-massive PE, early thrombolysis significantly reduced mortality as compared to treatment with heparin (2.7% vs. 11% in the heparin group; *p* = 0.033), whereas this effect was not seen in the female sex even on multivariate analysis [64]. However, this requires further exploration, as some prospective multicenter studies concluded that thrombolytic therapy is equally safe and beneficial in both sexes [64]. Pribish et al., in their single-center study on 2000 patients, found that despite the differences in comorbidities and presenting symptoms, management in terms of the need for intubation, vasopressors, IVC filters, ECMO, and AC regimen on discharge were similar among both sexes [20]. Similarly, Barrios et al. in their analysis of 2000 patients from a Spain-based registry, found similar rates of treatment with IVC filters and thrombolytic therapy among both sexes [15]. However, in contrast to this, from a registry-based study of 1400 patients in Tokyo, Tanabe et al. found that despite the higher incidence of severe and massive PE in women (14.6% vs. 9.2%; *p* = 0.0002), a statistically lower number of IVC filters were used in women (31.9% vs. 37.3%; *p* = 0.029) without a significant difference in proportions of invasive therapies like thrombolysis, catheter treatment, and surgeries in the female sex [21]. Despite the differences in incidence and clinical features, analysis of 371 patients from the EINSTEIN-PE study, which evaluated the use of rivaroxaban for the management of symptomatic PE, showed no sex-based differences in clot resolution at 3 weeks after treatment [65]. Menendez et al. conducted a retrospective follow-up study of 102 patients with PE who underwent serial perfusion scans at the time of diagnosis, at 7–10 days, and at 6 months [66]. Interestingly, the investigators found sex to be an independent predictor of clot size at 7–10 days. Particularly, female sex was found to be a significant risk factor of a larger clot size, and thereby slower resolution at the 7–10 day interval, among other risk factors. However, clot size at 7–10 days was found to be the only significant predictor of the size of the residual defect at 6-month follow-up.

Based on these studies, while we could say that the overall management of PE is similar in both sexes, there is a suggestion based on the National Inpatient Sample that thrombolytic therapy and IVC filters are less likely to be offered to women. 

### 3.6. Prognostication

There are several validated prognostic models, including the Pulmonary Embolism Severity Index (PESI), simplified PESI (sPESI), and European Society of Cardiology (ESC) model, which estimate the risk of mortality in patients with acute PE. Echocardiogram, troponin, and NT-proBNP are useful to identify the presence of RV dysfunction and stratify intermediate-high and intermediate-low risk PE [67,68]. Based on a study in Tuscany, sPESI score is better at predicting early mortality risk in females and as compared to males [67]. They also found that females with a sPESI score <2 had a significantly lower risk of death [67]. Another multicenter study in Tuscany found that despite seeing no difference in sPESI between the sexes, as per the 2008 ESC prognostic score, females were more likely to be categorized at high or intermediate risks as compared to males (81.5% vs. 71.5%; *p* = 0.0159) [69]. Berghaus et al. found that RV dysfunction was significantly more frequent in patients with central clots on CTPA and women taking oral contraceptive pills (*p* = 0.003) [31]. Keller et al. described other risk stratification and prognostic markers and found that RV dysfunction, cardiac troponin, sPESI, Bova score, and 2014 ESC algorithm predicted adverse outcomes in normotensive female patients, while tachycardia, hypoxia, NT-pro-BNP, and modified FAST scores predicted adverse outcomes in both sexes [22]. However, despite the difference in prognostic markers among sexes, 30-day adverse outcomes did not show a significant difference [22]. In a separate study, after multiple regression analysis, high levels of NT-proBNP and cardiac troponins did not reach statistical significance as a predictor of RV dysfunction [31]. This highlights the need of adjusting age- and sex-specific cut-offs for each of these biomarkers to increase their predictive values.

### 3.7. Short-Term Outcomes

#### 3.7.1. Complications

Recurrent pulmonary embolism and major bleeding have been frequently studied as major complications of acute PE. A California-based study identified that the male sex is associated with a significantly higher risk of recurrent PE [RR = 1.3, 95%CI: 1.0–1.6] as compared to female sex [70]. Of note, for African-American and Hispanic women, the rate of recurrent PE was found to be higher as compared to their Caucasian counterparts (*p* < 0.02) [70]. Multiple studies have found significantly higher rates of major bleeding in women as compared to men [15,22,66]. Keller et al. also found that major bleeding was a significant predictor of all-cause mortality in women [22]. Despite finding similar results of higher association of female sex with major bleeding and fatal PE, a study based on the RIETE registry demonstrated a loss of these endpoints on multivariate analysis [71]. Similarly, a separate single-center study showed no sex-based differences in the rate of major bleeding, readmissions, and recurrent PE at 90 days [20]. A Serbian based study also found equal incidence of bleeding despite the use of thrombolytics in approximately 60% of both sexes [42]. Using the data from EINSTEIN-PE study, Wiegers et al. found similar rates of clot resolution in both sexes after being treated with AC and suggested that clot resolution cannot account for the differences in recurrence rate of PE [65]. It can be argued that these complications are due to different patient characteristics and treatment choices with sex only acting as a confounding factor. This would warrant further studies looking into sex-specific differences in thrombosis and bleeding risk factors and comparing them with different treatment modalities to conclude whether a true difference exists. It could then guide us with sex-specific management to reduce complications, if indicated.

#### 3.7.2. Short-Term Mortality

Several studies evaluating short-term mortality in women have found no sex-based differences in in-hospital and 30-day all-cause mortality after a diagnosis of PE, despite sex-specific differences in prognosis predicted by risk stratification models [15,20,22,72]. Pribish et al., in their study of approximately 2000 patients with acute PE, of which half were women, found no sex-specific differences in in-hospital mortality despite differing comorbidity profiles and PE presentation between the sexes [20]. Despite no differences in all-cause mortality observed in previously published reports, Tanabe et al., in their Japanese registry-based study of 1428 patients with acute PE, observed a significantly higher 30-day PE-related mortality in women compared to men (mortality in women 5.0% vs. 2.8% in men; *p* = 0.043) [21]. It is possible that this was secondary to a significantly higher number of massive PE in women (women 14.6% vs. men 9.2%; *p* = 0.0002) and fewer women receiving IVC filters in this study population (women 31.9% vs. men 37.3%; *p* = 0.029) [21]. Notably, Barrios et al., in their study of nearly 2100 patients with acute PE, found female sex to be an independent predictor of both PE-related mortality (adjusted OR 1.85; 95%CI: 1.02–3.33; *p* = 0.04) and all-cause mortality (adjusted OR 1.56; 95%CI: 1.07–2.28; *p* = 0.02) only in hemodynamically stable patients, although when the entire study population was examined there was no difference in 30-day all-cause mortality between the sexes [15]. Sex-based differences in survival have also been studied in relation to institution of pulmonary embolism response team (PERT), wherein women had a lower survival to discharge rate in the pre-PERT era compared to men (women 91.5% vs. men 95%; *p* = 0.04), and in the post-PERT, no sex-based differences in survival was observed (women 93.1% vs. men 94.5%; *p* = 0.33) [20].

### 3.8. Long-Term Outcomes

#### 3.8.1. CTEPH

Chronic thromboembolic pulmonary hypertension (CTEPH) is a complication caused by multiple chronic pulmonary emboli which eventually lead to increased pressures in the pulmonary vascular leading to right heart failure [73]. Barco et al. investigated the European CTEPH registry and found a treatment discrepancy among both sexes [74]. More men underwent pulmonary endarterectomy (PEA) as compared to women (65% vs 54%; ARD (absolute risk difference), −11.0%; 95%CI: −18.2 to −3.6) and women were subjected to fewer additional cardiac procedures like coronary artery bypass graft surgery (0.5% vs. 9.5%; ARD, −9.0%; 95%CI: −13.6 to −4.9) [74]. Despite this difference, female sex was associated with higher long-term survival, despite having similar short-term mortality among both sexes [74]. Significant differences among sexes were also found in studying the prognosis of patients with CTEPH based on their hemodynamics. In females, the mean right atrial pressure and mixed venous oxygen saturation were found to be independent predictors of event-free survival in both before and after the acute vasoreactivity testing, while in males, it was the change in SvO2 (ΔSvO_2_) [75]. Researchers have investigated the use of cardiopulmonary exercise testing (CPET) to assess the disease severity, which can potentially be the non-invasive surrogate for the gold standard of right heart catheterization. On investigating the sex-related differences of CPET indices, Chen et al. found that the correlation of different CPET parameters for gas exchange efficiency with PVR was different among the sexes and concluded that these measurements could help in estimating the prognosis of CTEPH [76].

It is also interesting to note that the differences in incidence of CTEPH between sexes can vary based on ethnicity and genetics. Investigators in Japan found a significant difference in the female-to-male ratio of CTEPH between USA (0.7) and Japan (2.1), despite similar incidence of DVT among sexes [77]. They concluded that women with CTEPH had a positive correlation with HLA-B*5201, which was unrelated to DVT [77]. Based on this Shigeta et al. compared the clinical characteristics of females with CTEPH in Japan and found that overall, women had fewer acute embolic episodes, (34.0 vs. 70.2%; *p* < 0.001), lower prevalence of DVT (31.1 vs. 55.3%; *p* = 0.005), and lower surgical mortality (0 vs. 40%; *p* = 0.0098) as compared to males [78]. Among women, HLA-B*5201-positive genotype had significantly lower incidence of DVT (13.5 vs. 42.3%; *p* = 0.0036) and non-type 1 disease (13.3 vs. 48%; *p* = 0.02) as compared with HLA-B*5201-negative females [78].

#### 3.8.2. Long-Term Mortality

Sex-specific long-term mortality data is limited in the current literature. However, studies show increased long-term mortality (median follow-up ranging 15 to 21 months) in patients who suffered a PE compared to those who did not [79,80]. Siddique et al. evaluated long-term mortality after PE over a 10-year period and found men to have lower survival rates compared to women, although in both sexes, survival declined with advancing age [81].

### 3.9. Special Focus

#### 3.9.1. PE in Pregnancy

Venous thromboembolism has been found to be ten times more common in the pregnant population compared with non-pregnant women, with an estimated incidence of 1 in 1000, with the risk of PE being highest in the immediate postpartum period [82,83]. However, a recent study by Sun et al. on 1400 women found that PE was more commonly found in post-partum and non-pregnant women compared to pregnant women [84]. Due to the known adverse effects of radiation associated with CT of the chest in these patients, including increased risk of breast cancer even in the post-partum period [85], more population-specific screening cut-offs with D-dimer should be used. Considering the normal trend of increasing D-dimer levels during pregnancy [86], Zhang et al. concluded that by using the D-dimer cut off of 800 ng/mL, the sensitivity of detecting a PE was found to be 100% (with a specificity of 25.26%) as compared to D-dimer value of 1000 ng/mL, which had a sensitivity of 96.67%, hence increasing the number of patients excluded from suspected PE from 9.6 to 18.4% [87]. They also noted a significantly higher risk of PE in patients with known thrombophilia [87]. A study of 2300 women in Egypt also showed that D-dimer testing was 100% sensitive for PE, but CT was required to rule in the diagnosis [82].

After risk-stratifying with D-dimer, V/Q SPECT (ventilation/perfusion single photon emission computed tomography), a nuclear medicine scan with significantly lower radiation exposure to the patient and her fetus was found to have a high negative predictive value [88]. As technology has evolved, modern CT pulmonary angiography techniques expose patients to 3–4 mGy of radiation, which only causes a lifetime increase of cancer risk by a factor of 1.0003–1.0007 [86]. Hence, the 2019 ESC guidelines validate the use of NM scan or CTPA in high-risk women with positive D-dimer [86]. The possibility of using CTPA safely for diagnosis even in pregnant women is a welcome addition, considering that a recent clinical trial (SRCTN21245595) by Goodacre et al. found it difficult to use clinical features, validated diagnostic predictive tools like Well’s criteria, and simplified Geneva score and biomarkers to accurately select pregnant and postpartum women with a suspected PE for diagnostic imaging [89].

It is also important to highlight the possible pre-operative risk factors for post-partum PE in women undergoing cesarian section. Using the Taiwan database, Wang et al. concluded that chronic heart disease, systemic lupus erythematosus, post-partum hemorrhage, post-partum blood transfusion, and post-partum infection were significantly associated with PE within 40 days of cesarean section [90]. From their analysis of the Swedish registries, Ros et al. found that PE was seven times more likely in pregnant women with preeclampsia, with a relative risk of 22.6 compared to non-pregnant women (relative risk: 3), with higher risks being towards late pregnancy, at delivery, and in puerperium [91]. They also concluded that multiple births and cesarean deliveries were associated with higher risks of PE in pregnancy [91].

#### 3.9.2. PE in COVID-19

The COVID-19 pandemic caused by SARS-CoV-2 virus has resulted in 6,331,208 deaths across the world as of 12 June 2022 [92]. Pulmonary embolism is one of the many comorbidities in patients with COVID-19, with the most recent meta-analysis showing a cumulative incidence of 21%; 95%CI: 18−24%; *p* < 0.001 [93]. Even though a systematic review of sex-based differences in PE in COVID-19 was not possible due to the paucity of sex-based studies in this field, we aimed to highlight the main findings that would aid in improved understanding of PE in patients with COVID-19.

A multicenter study in France found that the male sex [OR: 1.83, 95%CI: 1.19–2.89; *p* = 0.009] was significantly associated with PE occurrence in both univariate and multivariate analysis [94]. A USA-based retrospective study also found increased incidence of PE in men (OR: 1.74; 95%CI: 1.1, 2.8; *p* = 0.02); along with an association of PE with smoking (OR: 1.86; 95%CI: 1.0, 3.4; *p* = 0.04) [95]. However, the French study did not find a significant association of smoking or higher age with PE in COVID-19 [94]. Based on the risk factors of PE seen in the general population, the increased incidence of PE in men with COVID-19 could either be due to a higher incidence of smoking in men or it could be a true association due to different genetic makeup. The evidence points more towards an independent sex-based propensity of increased PE incidence in men, which can be further supported by other studies conducted in Iran [96]. On the other hand, some single-center studies did not show a risk of PE in COVID-19 in either sex [97]. This could possibly be attributed to the low power or the fact that COVID-19 itself is seen more commonly in men, with a pooled prevalence of 55.00 (51.43–56.58; I[2] = 99.5%; *p* < 0.001) [98], which leads to a false positive association of the male sex with PE in COVID-19. The unfavorable outcomes in male sex persisted with a 45% increase in deaths in men as compared to 14% increase of deaths in women in an Italian study of PE-related mortality in patients with COVID-19.

## 4. Future Directions

Currently, the available literature on pulmonary embolism is extensive, but unanswered questions remain for future research. From a presentation standpoint, more dedicated research is needed to explore the severity of presentation in women and why women present with higher NT-proBNP levels despite normal RV size. From a diagnostic perspective, evaluating whether the diagnostic accuracy of CTPA differs based on sex needs to be explored. Despite a higher overall age- and sex-adjusted incidence of PE in men, the risk of PE in older women supersedes that of age-matched men. It is unclear whether this increased risk stems from factors such as hormone replacement therapy or if there is a true sex-based predilection for older women. This is clinically relevant because if the latter was true, healthcare professionals would need to be more vigilant of symptoms in older women. Another interesting finding is hemoptysis, which is a more common presenting symptom in men with PE compared to women. Exploring whether this finding is associated with the increased prevalence of cancer in men with PE is important. 

## Data Availability

Data sharing not applicable.

## Supplementary Materials

The following supporting information can be downloaded at: https://www.mdpi.com/article/10.3390/jcdd9080234/s1. Table S1: PRISMA checklist.
