# Supplementary material for: Pulmonary Embolism in Women: A Systematic Review of the Current Literature
Source: J Cardiovasc Dev Dis. 2022 Jul 25;9(8):234. doi: 10.3390/jcdd9080234 (PMC9330775; doi:10.3390/jcdd9080234)
Supplement: Supplementary file 1 [file jcdd-09-00234-s001.zip › jcdd-1807038-supplementary.pdf]

Supplementary Material

Table S1: PRISMA checklist

Section and Topic	Item #	Checklist item	Location where item is reported
**TITLE**			
Title	1	Identify the report as a systematic review.	1
**ABSTRACT**			
Abstract	2	See the PRISMA 2020 for Abstracts checklist.	2
**INTRODUCTION**			
Rationale	3	Describe the rationale for the review in the context of existing knowledge.	3-4
Objectives	4	Provide an explicit statement of the objective(s) or question(s) the review addresses.	4
**METHODS**			
Eligibility criteria	5	Specify the inclusion and exclusion criteria for the review and how studies were grouped for the syntheses.	5
Information sources	6	Specify all databases, registers, websites, organisations, reference lists and other sources searched or consulted to identify studies. Specify the date when each source was last searched or consulted.	4-5
Search strategy	7	Present the full search strategies for all databases, registers and websites, including any filters and limits used.	4-5, Figure 1
Selection process	8	Specify the methods used to decide whether a study met the inclusion criteria of the review, including how many reviewers screened each record and each report retrieved, whether they worked independently, and if applicable, details of automation tools used in the process.	5
Data collection process	9	Specify the methods used to collect data from reports, including how many reviewers collected data from each report, whether they worked independently, any processes for obtaining or confirming data from study investigators, and if applicable, details of automation tools used in the process.	5

Section and Topic	Item #	Checklist item	Location where item is reported
Data items	10a	List and define all outcomes for which data were sought. Specify whether all results that were compatible with each outcome domain in each study were sought (e.g. for all measures, time points, analyses), and if not, the methods used to decide which results to collect.	4-5
	10b	List and define all other variables for which data were sought (e.g. participant and intervention characteristics, funding sources). Describe any assumptions made about any missing or unclear information.	4-5
Study risk of bias assessment	11	Specify the methods used to assess risk of bias in the included studies, including details of the tool(s) used, how many reviewers assessed each study and whether they worked independently, and if applicable, details of automation tools used in the process.	N/A
Effect measures	12	Specify for each outcome the effect measure(s) (e.g. risk ratio, mean difference) used in the synthesis or presentation of results.	5
**RESULTS**			
Study selection	13a	Describe the results of the search and selection process, from the number of records identified in the search to the number of studies included in the review, ideally using a flow diagram.	Figure 1
	13b	Cite studies that might appear to meet the inclusion criteria, but which were excluded, and explain why they were excluded.	Figure 1
Study characteristics	14	Cite each included study and present its characteristics.	N/A
Risk of bias in studies	15	Present assessments of risk of bias for each included study.	N/A
Results of individual studies	16	For all outcomes, present, for each study: (a) summary statistics for each group (where appropriate) and (b) an effect estimate and its precision (e.g. confidence/credible interval), ideally using structured tables or plots.	6-23
Results of syntheses	17a	For each synthesis, briefly summarise the characteristics and risk of bias among contributing studies.	N/A
	17b	Present results of all statistical syntheses conducted. If	N/A

Section and Topic	Item #	Checklist item	Location where item is reported
		meta-analysis was done, present for each the summary estimate and its precision (e.g. confidence/credible interval) and measures of statistical heterogeneity. If comparing groups, describe the direction of the effect.	
**DISCUSSION**			
Discussion	18a	Provide a general interpretation of the results in the context of other evidence.	6-23
	18b	Discuss any limitations of the evidence included in the review.	22-23
	18c	Discuss any limitations of the review processes used.	22-23
	18d	Discuss implications of the results for practice, policy, and future research.	23
**OTHER INFORMATION**			
Registration and protocol	19a	Provide registration information for the review, including register name and registration number, or state that the review was not registered.	N/A
	19b	Indicate where the review protocol can be accessed, or state that a protocol was not prepared.	N/A
	19c	Describe and explain any amendments to information provided at registration or in the protocol.	N/A
Support	20	Describe sources of financial or non-financial support for the review, and the role of the funders or sponsors in the review.	24
Competing interests	21	Declare any competing interests of review authors.	24
Availability of data, code and other materials	22	Report which of the following are publicly available and where they can be found: template data collection forms; data extracted from included studies; data used for all analyses; analytic code; any other materials used in the review.	N/A

PRISMA: Preferred Reporting Items for Systematic Reviews and Meta-Analyses
